# Shock and Awe: Successful Revascularization with Intravascular Lithotripsy in Recurrent ST-Elevation Myocardial Infarction Secondary to Stent Under-Expansion

**DOI:** 10.14797/mdcvj.1257

**Published:** 2023-08-17

**Authors:** Pramod Kumar Ponna, Akhilesh Gonuguntla, Ramya Krishna Botta, Sruthi Kotaru, Tim A. Fischell, Adnan Alexander Kassier, Yashwant Agrawal

**Affiliations:** 1 Louisiana State University Health Sciences Center, Shreveport, Louisiana, US; 2 Johns Hopkins University, Baltimore, Maryland, US; 3 Vanderbilt University Medical Center, Nashville, Tennessee, US; 4 Western Michigan University Homer Stryker MD School of Medicine, Kalamazoo, Michigan, US; 5 Mercy Hospital, Springfield, Missouri, US; 6 Banner Desert Medical Center, Mesa, Arizona, US

**Keywords:** intravascular lithotripsy, calcified coronary artery, under-expanded stent, ST-elevation myocardial infarction

## Abstract

We report a case of recurrent ST-segment elevation myocardial infarction (STEMI) due to a previously implanted under-expanded stent with in-stent thrombosis refractory to traditional interventional techniques. We underscore the utility of bail-out shockwave intravascular lithotripsy to tackle previously under-expanded stents in this acute setting.

## Introduction

Percutaneous coronary intervention on heavily calcified coronary plaque is an interventional challenge associated with a lower success rate and a higher risk of complications. Heavy calcification leads to incomplete stent expansion with subsequent stent apposition and failure. Stent under-expansion can predispose patients to acute stent thrombosis and premature in-stent restenosis.^1^ Traditionally, atherectomy is used to treat high-risk lesions with underlying stent under-expansion. However, this may be technically difficult since the device is physically impeded by the stent in situ^2^ and may not address issues of deeper vessel wall calcification. Intravascular lithotripsy (IVL) is an attractive alternative to circumferentially disrupt severely calcified coronary lesions contributing to significant luminal gain. It mitigates the risk of stent under-expansion, in-stent stenosis, and thrombosis.

## Case

A 54-year-old male with coronary artery disease and multiple recent percutaneous coronary interventions (PCIs), hypertension, hyperlipidemia, and tobacco use presented with severe chest pain. Fourteen days prior, he had undergone PCI at an outlying hospital, where a stent was implanted in his right coronary artery (RCA) for unstable angina (with 80% proximal RCA stenosis).

The angiogram of the first intervention was unavailable to our facility. However, the interventional details were as follows. A 3.5- x 38-mm drug-eluting stent was deployed in the proximal-mid RCA, which was post-dilated using a 4.0- x 12-mm noncompliant balloon at 24 atmospheres (atm). Intravascular imaging after post-dilating the stent revealed a minimal stent area of 4.3 mm.^2^ The patient was discharged on aspirin 81 mg daily, ticagrelor 90 mg twice daily, and rivaroxaban 2.5 mg twice daily as his antiplatelet regimen.

**Figure 1:**
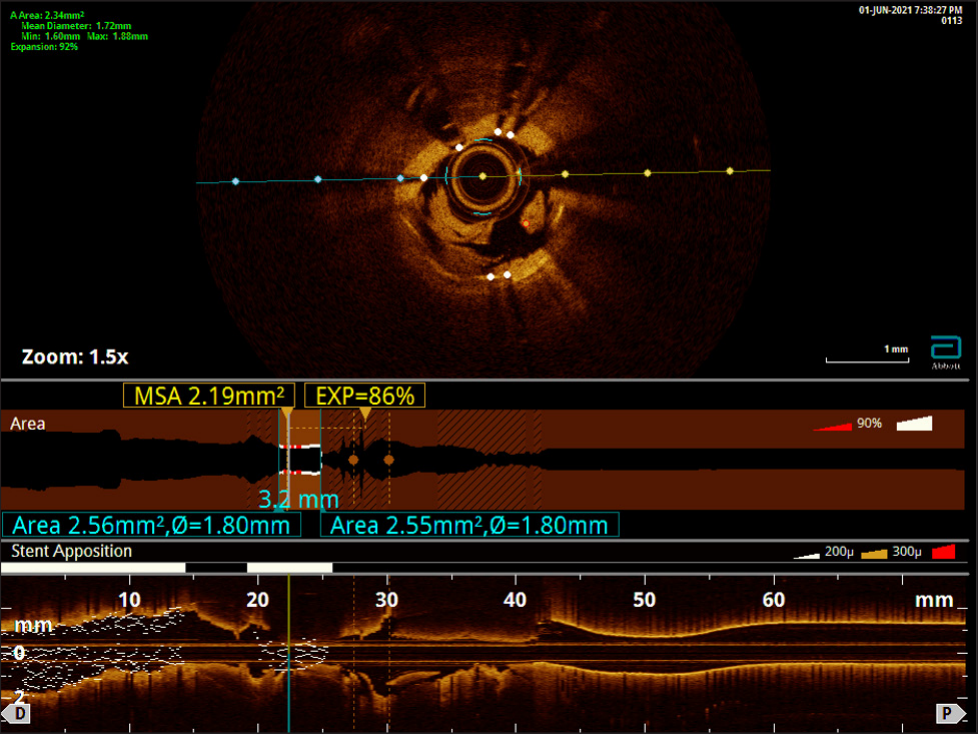
Optical coherence tomography of the right coronary artery showed significant calcification along with severely under-expanded and malapposed stent.

Four days after this procedure, the patient presented to our facility with acute inferior STEMI due to acute stent thrombosis (Video 1). Balloon angioplasty effectively restored blood flow to Thrombolysis in Myocardial Infarction (TIMI) risk score 3. Optical coherence tomography (OCT) showed significant calcification and a severely under-expanded and malapposed stent (Figure 1). Percutaneous coronary intervention was performed with a 3.5-mm noncompliant balloon at 24 atm and then with a 3.5-mm chocolate balloon (Medtronic). Final angiographic findings revealed 30% to 40% residual stenosis with TIMI-3 flow (Video 2). Shockwave angioplasty was considered; however, the use of Shockwave in a recently implanted stent in the setting of subacute stent thrombosis was clearly off-label, and therefore Shockwave angioplasty was not performed. We decided to continue the patient on his antiplatelet regimen of aspirin 81 mg daily, ticagrelor 90 mg twice daily, and rivaroxaban 2.5 mg twice daily.

**Video 1:** Coronary angiography revealed 100% occluded right coronary artery stent causing the patient’s inferior ST-elevation myocardial infarction; see also at https://youtube.com/shorts/ee7qbm-a8G4

**Video 2:** Final angiographic findings revealed residual 30% to 40% stenosis with Thrombolysis in Myocardial Infarction-3 flow in the right coronary artery; see also at https://youtube.com/shorts/iqwHACzyv4A

Ten days after the procedure, the patient presented with recurrent inferior STEMI due to acute stent thrombosis (Video 3). After balloon angioplasty to restore TIMI-3 flow, intravascular ultrasound (IVUS) imaging was performed, confirming heavy circumferential calcification of the RCA with an under-expanded and malapposed stent encased in calcium (Video 4). The distal vessel reference was 3.5 mm, whereas the in-stent expansion was 1.5 mm.

**Video 3:** Coronary angiography revealed 100% occluded right coronary artery stent causing the patient’s recurrent inferior ST-elevation myocardial infarction; see also at https://youtube.com/shorts/ACLrnSVUODc

**Video 4:** Intravascular ultrasound of the right coronary artery revealed severely calcified lesion with under-expanded and malapposed stent; see also at https://youtube.com/shorts/8oIxN4q0rKo

Given these findings, bail-out IVL was performed successfully with a 3.5- x 12-mm Shockwave C2 Coronary IVL balloon (Shockwave Medical, Inc.) at 4 atm (80 pulses). This yielded the lesion angiographically, and the IVUS catheter revealed fragmentation of the calcification and expansion of the stent (Video 5). This was followed by high-pressure noncompliant balloon inflation and eventual stent release and expansion, resulting in 0% residual stenosis and TIMI-3 flow in the RCA (Video 6). The patient tolerated the procedure well without any complications. The patient was seen at 6-, 12-, and 18-month follow-up visits. He remained asymptomatic from a cardiac standpoint on aspirin 81 mg and ticagrelor 90 mg twice daily as an antiplatelet regimen, with no recurrent events since the last procedure.

**Video 5:** Intravascular ultrasound revealed fragmentation of the calcification and expansion of the stent; see also at https://youtube.com/shorts/HeJhQfgemY4

**Video 6:** Final angiographic findings revealed residual 0% stenosis with Thrombolysis in Myocardial Infarction-3 flow in the right coronary artery; see also at https://youtube.com/shorts/y1svDHpX4G0

## Discussion

Coronary artery calcification is a challenging entity associated with poor long-term outcomes if not adequately treated. Calcified lesions predispose to device-related adverse events such as stent under-expansion and subsequent failure.^1^ Conventional lesion preparation strategies utilize high-pressure non-compliant balloons, modified balloons (cutting/scoring), or atheroablative technology to improve stent deployment in coronary calcifications. However, these interventions are associated with a high risk of strategy failure and coronary artery injury (dissection/perforation).^3^ Shockwave IVL (S-IVL) is a novel calcium modification device that emits sonic pressure waves that circumferentially fracture superficial and deep coronary calcific lesions to improve vessel compliance and facilitate stent implantation. The Disrupt CAD I-IV studies emphasized the high procedural success rates and safety of S-IVL in lesion preparation of severely calcified de novo coronary stenoses, paving the way for US Food and Drug Administration approval in these patients.^4,5^

Few studies have explored the off-label use of S-IVL in stent under-expansion and failure.^6,7^ Data from a multicenter French registry (n = 65) revealed a relatively lower angiographic success rate with in-stent use (73.1%) compared to use in native coronary arteries (94.9%) but similar clinical outcomes in their cohort.^5^ CRUNCH registry data (n = 70) showed a device success rate of 92.3% with no complications or major adverse cardiac events in their cohort of patients with stent under-expansion due to coronary calcification.^7^ In a similar cohort, SMILE registry data (n = 39) exhibited a device success rate of 87.1%, with only one patient with a complication of nonfatal periprocedural STEMI secondary to balloon rupture. All other patients had no complications or major adverse cardiac events.^8^ In addition, there appears to be a common concern among researchers on the effect of acoustic energy on stent drug-polymer coating, potentially increasing the risk of future in-stent lesions. S-IVL has a high safety index and impressive efficacy for in-stent lesions. However, the role of the technology in modifying coronary calcium in acute coronary syndrome-related stent under-expansion is unclear.

There is no clear direction on the optimal revascularization strategy for acute coronary syndrome related to under-expanded stents secondary to calcified plaques. The data on the utility of S-IVL in this setting is limited to case reports as a bail-out technique when conventional methods fail. Salazar et al. managed STEMI associated with stent under-expansion with S-IVL (4 atm, 60 pulses). They discussed the drawback of traditional calcium modifying devices physically impeded by the stent metallic matrix during lesion preparation, preventing rapid, crucial luminal gain.^2^ Dimitriadis et al. used S-IVL (4 atm, 30 pulses) for non-STEMI in a similar patient and successfully treated the patient with no complications.^9^ Agrawal et al. used S-IVL (3-6 atm, 80 pulses) for non-STEMI in a similar but unstable patient in cardiogenic shock with Impella support.^10^ All three authors performed intravascular imaging by OCT or IVUS to assess the calcified coronary arteries. Intravascular imaging can help provide more information on stent under-expansion and help identify associated incomplete stent apposition, absence of strut endothelization, or stent fracture. Moreover, both cases highlighted the safety and efficacy benefits of the off-label use of S-IVL to manage stent failure in calcified coronaries. Importantly, S-IVL is probably best reserved for lesions with stent under-expansion related to dense, deep, circumferential, or nearly circumferential calcification.

**Figure 2:**
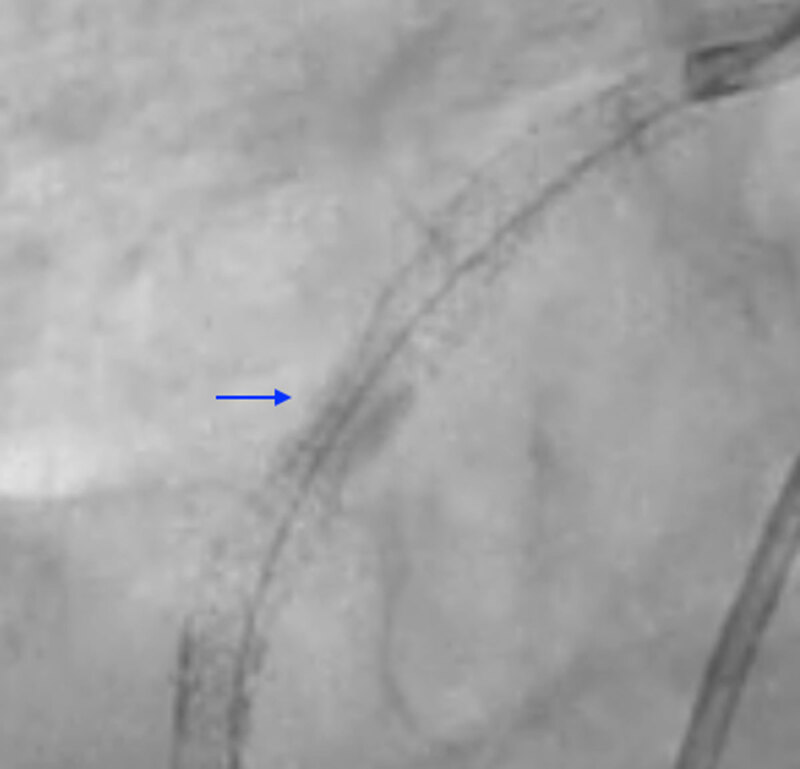
Dry-cine of the right coronary artery stent pre-intravascular lithotripsy.

**Figure 3:**
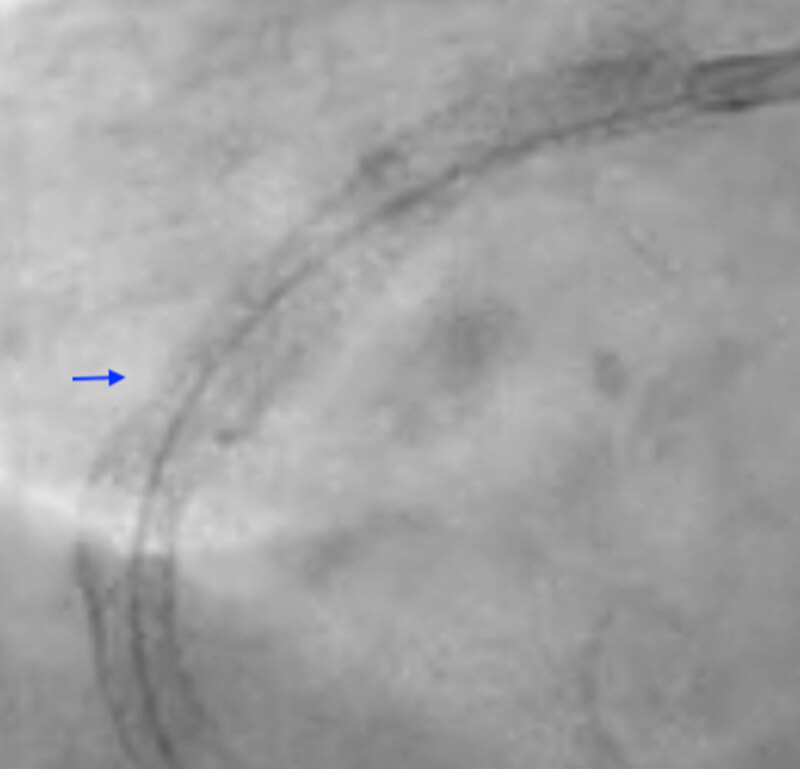
Dry-cine of the right coronary artery stent post-intravascular lithotripsy revealing stent-release.

Our case highlights the successful utilization of S-IVL for “stent-release” (Figures 2 and 3) in subacute stent thrombosis leading to STEMI from a severely under-expanded stent. However, large-scale studies are required to better delineate the utility of S-IVL in this setting.

## Conclusion

S-IVL is a safe modality to circumferentially disrupt severely calcified coronary lesions to improve vessel compliance and promote luminal gain before angioplasty. Further studies are required to establish the safety and efficacy of S-IVL in acute coronary syndrome patients with stent under-expansion or failure.
